# Analysis of the anti-PCV2 mechanism of *Lactobacillus acidophilus* based on non-target metabolomics and high-throughput molecular docking

**DOI:** 10.3389/fmicb.2024.1416235

**Published:** 2024-05-27

**Authors:** Zhigang Cao, Xiaoya Ling, Abdul Haseeb, Panpan Sun, Hua Zhang, Wei Yin, Kuohai Fan, Huizhen Yang, Zhenbiao Zhang, Jia Zhong, Yaogui Sun, Na Sun, Hongquan Li

**Affiliations:** ^1^Shanxi Key Laboratory for Modernization of TCVM, College of Veterinary Medicine, Shanxi Agricultural University, Jinzhong, Shanxi, China; ^2^Laboratory Animal Center, Shanxi Agricultural University, Jinzhong, Shanxi, China

**Keywords:** *Lactobacillus acidophilus*, PCV2, Cap, Rep, non-target metabolomics, high-throughput molecular docking

## Abstract

Our previous studies have revealed that *L. acidophilus* possesses inhibitory effects on PCV2 proliferation *in vivo*, although the underlying mechanisms remain elusive. Probiotics like *L. acidophilus* are known to exert antiviral through their metabolites. Therefore, in this study, non-targeted metabolomics was used to detect the changes in metabolites of *L. acidophilus* after 24 h of proliferation. Subsequently, high-throughput molecular docking was utilized to analyze the docking scores of these metabolites with PCV2 Cap and Rep, aiming to identify compounds with potential anti-PCV2 effects. The results demonstrated that 128 compounds such as Dl-lactate were significantly increased. The results of high-throughput molecular docking indicated that compounds such as ergocristine, and telmisartan formed complexes with Cap and Rep, suggesting their potential anti-PCV2 properties. Furthermore, compounds like vitamin C, exhibit pharmacological effects consistent with *L. acidophilus* adding credence to the idea that *L. acidophilus* may exert pharmacological effects through its metabolites. These results will provide a foundation for the study of *L. acidophilus*.

## 1 Introduction

PCV2 serves as the fundamental pathogen responsible for porcine circovirus-associated disease (PCVAD) (Opriessnig et al., 2007). This virus possesses a single-stranded, circular DNA genome with a length of approximately 1.7 kb, characterized by a covalent bond. The diameter of the virus particles is about 15–20 nm, showing a symmetrical structure of the icosahedron. The entire PCV2 genome comprises 11 open reading frames (ORF), including ORF1, ORF2, ORF3, ORF4, etc. ORF1 and ORF2, oriented in opposite directions, are the two major ORFs in PCV2. ORF1 encodes replicase protein (Rep) comprised of 314 amino acid residues, while ORF2 encodes a 27.8 kDa capsid protein (Cap) comprised of 233–236 amino acid residues. These two proteins play a crucial role in viral replication, infection, and transmission (Hamel et al., 1998; Nawagitgul et al., 2002; Luo et al., 2018). PCV2 infection in pigs can lead to clinical symptoms such as dyspnea and abortion (Segalés et al., 2005) along with pathological changes like interstitial pneumonia (Allan et al., 1998), and alterations in the structure of intestinal flora (Niederwerder et al., 2016). Since its discovery in 1998 (Ellis et al., 1998), PCV2 has rapidly disseminated worldwide, resulting in significant economic repercussions for the swine industry. First reported in China in 2000, the virus has continued to pose challenges, with an increasing infection rate observed from 2004 to 2020, consistently exceeding 45% (Shuai et al., 2007; Huang et al., 2021). Currently, both in China and worldwide, PCV2 infection rates remain high. To prevent and control the spread of PCV2, a total number of 53 production enterprises in China have obtained the production approval number for the porcine circovirus vaccine according to the National Veterinary Drug Basic Information Database. While the use of PCV2 vaccines has shown some efficacy in controlling the multisystemic wasting syndrome in weaned piglets induced by PCV2, it has limitations. Factors such as the continuous variation of PCV2 strains (Rajkhowa et al., 2021) and the immunosuppression induced by the virus necessitate the exploration of additional prevention and control methods beyond vaccine immunization alone.

Our screening exertions have identified matrine as possessing an anti-PCV2 effect *in vitro* (Sun et al., 2015). Furthermore, the administration of 40 mg/kg matrine has proven to significantly inhibit PCV2 replication in mice and ameliorate pathological manifestations, such as virus-induced widening of the pulmonary septum (Sun et al., 2020). In our pursuit to explore the mechanisms of matrine against PCV2, a previous study revealed that intraperitoneal injection of 2 mg/mL matrine could enhance the proliferation of intestinal *L. acidophilus* (Cao et al., 2023). In a subsequent experiment involving PCV2-infected mice, the administration of *L. acidophilus* via gavage or enema at varying concentrations led to a significant reduction in the viral load of PCV2 in the liver and ileum compared to the blank control group. These results provide compelling evidence that matrine exerts its anti-PCV2 role by modulating intestinal *L. acidophilus* (Cao et al., 2023). However, the precise mechanism underlying this regulatory interaction is yet to be elucidated.

Studies have shown that *L. acidophilus* exhibits anti-viral effects against PCV2, Herpes Simplex-1 Virus (HSV-1), Vesicular Stomatitis Virus (VSV), H9N2, and other viruses (Gao et al., 2016; Cao et al., 2023; Elebeedy et al., 2023). A study by Lu et al. (2021) revealed that *Lactobacillus mucosae 1,025* and *Bifidobacterium breve CCFM1026* could inhibit the infection of influenza A virus in mice and this antiviral effect was attributed to its metabolite butyrate. Antunes et al. (2019) found that acetic acid produced by the metabolism of intestinal flora can regulate the type I interferon response of lung epithelial cells by activating the Gpr43 receptor, thereby protecting mice from RSV infection. Additionally, Nácher-Vázquez et al. (2015) discovered that the glucan produced by lactic acid bacteria has antiviral and immunomodulatory activity against the salmon virus. These studies collectively suggest that the potential anti-PCV2 effect of *L. acidophilus* might be linked to its metabolites.

To investigate whether the anti-PCV2 impact of *L. acidophilus* is associated with its metabolites, the conducted experiment analyzed alterations in the metabolites present in the culture supernatant of *L. acidophilus* after 24 h of proliferation *in vitro* using non-target metabolomics. High-throughput molecular docking was employed to screen for compounds that may possess anti-PCV2 effects, providing insights into the potential mechanism of *L. acidophilus* against PCV2.

## 2 Materials and methods

### 2.1 Compounds and *L. acidophilus*

MRS Broth (Batch number: M8540) was purchased from Beijing Solarbio Technology Co., Ltd. *L. acidophilus* was isolated and identified by the Shanxi Key Lab. for Modernization of TCVM.

### 2.2 Non-target metabolomics detection of metabolome changes in the supernatant of *L. acidophilus* culture medium

The *L. acidophilus* preserved at −80°C was streaked onto MRS solid medium and cultured inverted at 37°C for 48 h. A single colony from the aforementioned MRS solid medium was selected and inoculated into 5 mL of MRS liquid medium. The inoculated medium was placed on a shaker at 37°C and 220 rpm for 24 h. After centrifugation at 4000 g for 5 min, the supernatant of the culture was collected. *L. acidophilus* culture supernatant was subjected to Non-targeted metabolomics analysis by Shanghai Applied Protein Technology. Briefly, samples were prepared for HPLC–MS/MS analysis on a UPLC system (Agilent 1,290 Infinity UHPLC) according to the protocol.

### 2.3 UHPLC-Q-TOF MS conditions in non-target metabolomics

Analysis was performed using a UHPLC (1,290 Infinity LC, Agilent Technologies) coupled to a quadrupole time-of-flight (AB Sciex TripleTOF 6,600) in Shanghai Applied Protein Technology Co., Ltd. For HILIC separation, samples were analyzed using a 2.1 mm × 100 mm ACQUIY UPLC BEH Amide 1.7 μm column (waters, Ireland). In both ESI positive and negative modes, the mobile phase contained A = 25 mM ammonium acetate and 25 mM ammonium hydroxide in water and B = acetonitrile. The gradient was 95% B for 0.5 min and was linearly reduced to 65% in 6.5 min, and then was reduced to 40% in 1 min and kept for 1 min, and then increased to 95% in 0.1 min, with a 3 min re-equilibration period employed. The ESI source conditions were set as follows: Ion Source Gas1 (Gas1) as 60, Ion Source Gas2 (Gas2) as 60, curtain gas (CUR) as 30, source temperature: 600°C, IonSpray Voltage Floating (ISVF) ± 5,500 V. In MS acquisition, the instrument was set to acquire over the m/z range 60–1,000 Da, and the accumulation time for TOF MS scan was set at 0.20 s/spectra. In auto MS/MS acquisition, the instrument was set to acquire over the m/z range 25–1,000 Da, and the accumulation time for product ion scan was set at 0.05 s/spectra. The product ion scan was acquired using information-dependent acquisition (IDA) with a high sensitivity mode being selected. The parameters were set as follows: the collision energy (CE) was fixed at 35 V with ±15 eV; declustering potential (DP), 60 V (+) and − 60 V (−); exclude isotopes within 4 Da, candidate ions to monitor per cycle: 10.

### 2.4 High-throughput molecular docking screening of compounds that may have anti-PCV2

The metabolites in the culture supernatant of *L. acidophilus* that exhibited a substantially higher concentration following 24 h of *in vitro* proliferation were identified using non-targeted metabolomics. Subsequently, a small molecule library comprising these compounds was assembled. Molecular docking was performed using the UCSF DOCK6 program. The crystal structure of the Cap (PDB code: 5ZBO) and the Rep (PDB code: 2HW0) were used as receptors. The compounds in the small molecule library were docked with 5ZBO and 2HW0 respectively, to obtain the docking score of the compounds with the two receptors. The binding affinity of the compounds to the two receptors was evaluated by docking scoring. The screening process involved utilizing the PyMOL Molecular Graphics System (Version 2.5.7) to generate the binding pattern map and identify compounds that potentially exhibit anti-PCV2 effects in *L. acidophilus*.

### 2.5 Data analysis

After sum-normalization, the processed data were analyzed by R package (ropls), where it was subjected to multivariate data analysis, including Pareto-scaled principal component analysis (PCA) and orthogonal partial least-squares discriminant analysis (OPLS-DA). The 7-fold cross-validation and response permutation testing were used to evaluate the robustness of the model. The variable importance in the projection (VIP) value of each variable in the OPLS-DA model was calculated to indicate its contribution to the classification. Student’s t-test was applied to determine the significance of differences between two groups of independent samples. VIP > 1 and *p*-value <0.05 were used to screen significantly changed metabolites. Pearson’s correlation analysis was performed to determine the correlation between two variables.

## 3 Results

### 3.1 Quantitative analysis of *L. acidophilus* metabolites reveals extensive differential molecular

Following a 24-h culture period, the supernatant of the *L. acidophilus* culture medium was processed and analyzed using LC–MS/MS for untargeted metabolomics following the standardized protocol. Hydrophilic and hydrophobic molecules were analyzed using both positive and negative ionisation to cover various endogenous biochemical classes. In total, 1,449 metabolite peaks representing 647 and 775 differential metabolites in negative and positive ion modes respectively, were identified in the untargeted metabolomics analysis (Table 1).

**Table 1:** Statistics of identified metabolites.

Detection mode	Number of metabolites identified
Positive	775
Negative	674
Total	1,449

### 3.2 Identification of differential metabolites

**Figure 1:**
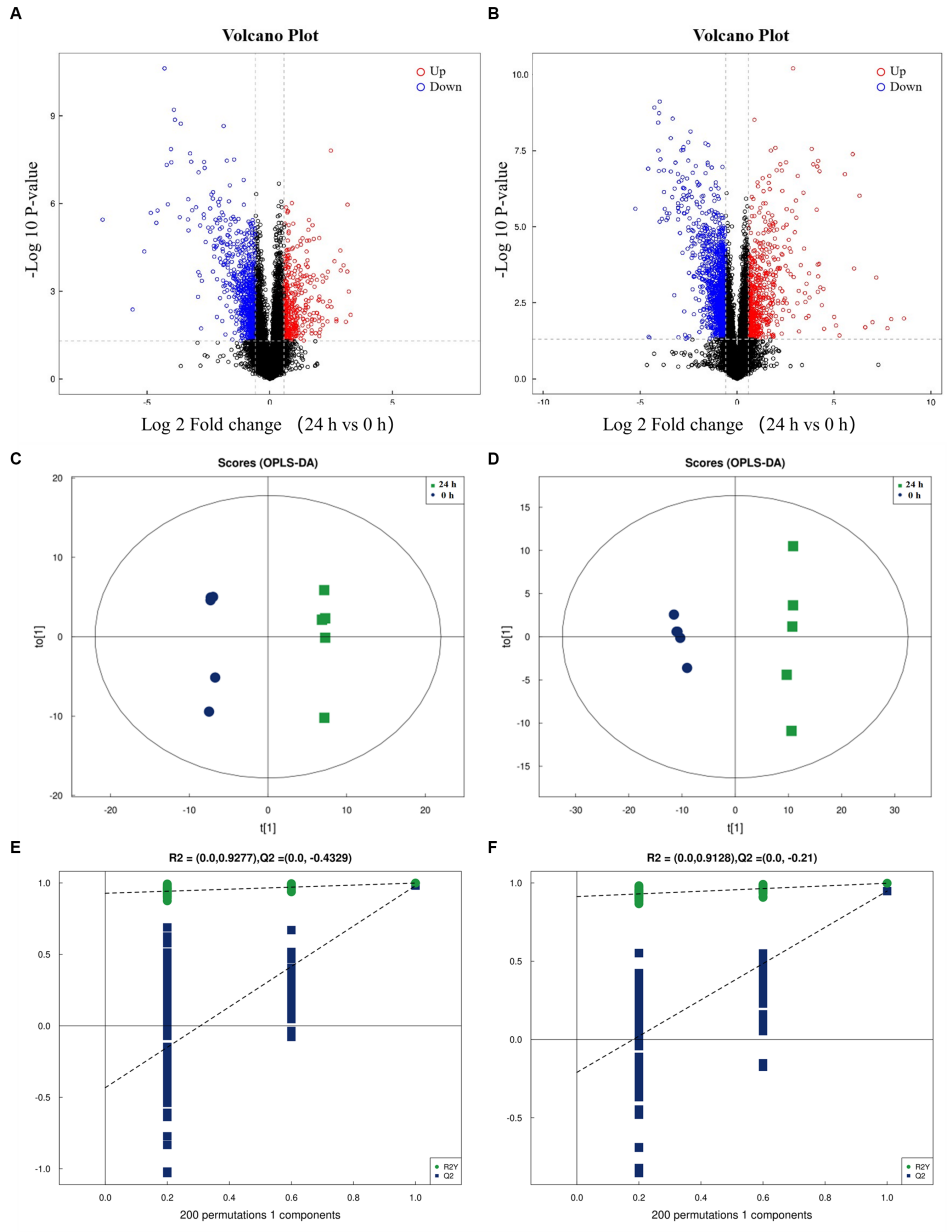
Screening of differential metabolites between the two groups. **(A)** Volcano diagram of metabolites of *L. acidophilus* supernatant in positive ion mode. **(B)** Volcano diagram of metabolites of *L. acidophilus* supernatant in negative ion mode. **(C)** OPLS-DA score plot of metabolites of *L. acidophilus* supernatant in positive ion mode. **(D)** OPLS-DA score plot of metabolites of *L. acidophilus* supernatant in negative ion mode. **(E)** Permutation test results of the OPLS-DA mode in positive ion mode. **(F)** Permutation test results of the OPLS-DA mode in negative ion mode.

Following a 24-h culture of *L. acidophilus*, the differential expression metabolites volcano map analysis revealed that, compared with the 0 h group, there were significantly up-regulated [FC (Fold change) > 1, *p* < 0.05] and down-regulated (FC < 1, *p* < 0.05) metabolites in the supernatant of *L. acidophilus* culture medium in 24 h group under positive (Figure 1A) and negative (Figure 1B) ion modes. The OPLS-DA score diagram of the 0 h group and the 24 h group under positive (Figure 1C) and negative (Figure 1D) ion modes showed clear differences between the two groups, indicating that there was a specific metabolome in the culture supernatant of *L. acidophilus* after 24 h proliferation *in vitro*. As a result of 7-fold cross-validation, the model exhibited no overfitting, which was evidenced by R^2^ and Q^2^ values of 0.9277 and − 0.4329 in positive ion mode (Figure 1E), and 0.9128 and − 0.21 in negative ion mode (Figure 1F), respectively. Response permutation testing 200 times further confirmed the model’s ability to account for the observed variations with *R*^2^Y values close to 1 in both positive and negative ion modes. The identification of differential metabolites was dependent on the OPLS-DA model’s VIP (Variable Importance in the Projection) being greater than 1 and the *p*-value being ≤0.05. As shown in Supplementary Table S1, 270 differential metabolites, including ergocristine, apicidin, penitrema, guanosine 5 'monophosphate, Trp-Arg, UDP-N-acetylmuraminate, telmisartan, and lopinavir metabolite were screened out after comparing the 0 h group with the 24 h group.

### 3.3 Analysis of trends in differential metabolite changes

**Figure 2:**
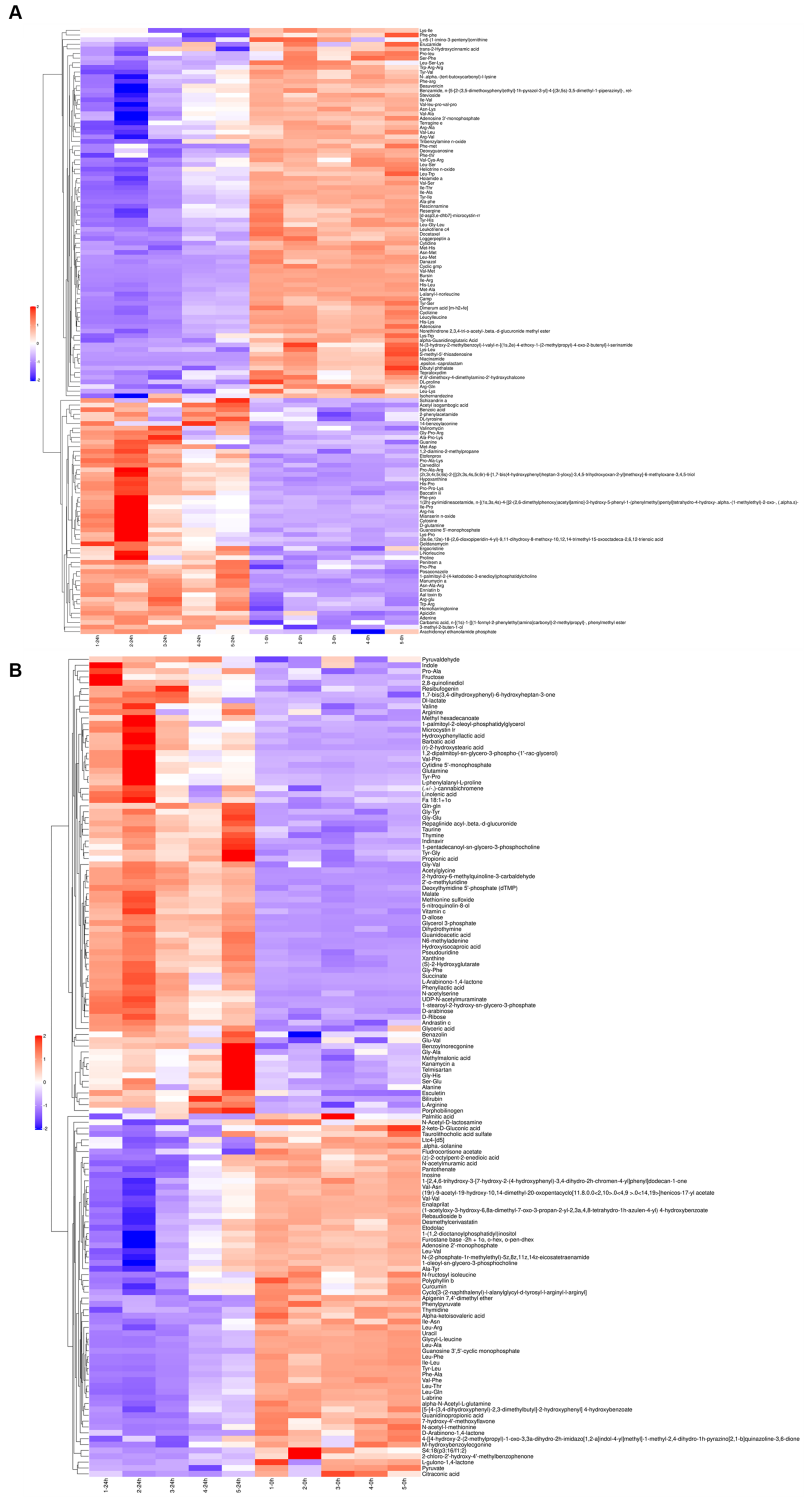
Cluster analysis for differential metabolites of *L. acidophilus* supernatant in positive **(A)** and negative **(B)** ion mode.

The differential metabolites in the positive (Figure 2A) and negative (Figure 2B) ion modes were analyzed by cluster analysis. After 24 h of *L. acidophilus in vitro* proliferation, the results demonstrated a significant up-regulation (*p* < 0.05) of 128 metabolites, including telmisartan, vitamin C, glutamine, carvedilol, Dl-lactate, succinate, penitrema, ergocristine, hypoxanthine, and arginine, in comparison with C 0 h. While 142 metabolites such as adenosine, niacinamide, cyclizine, thymidine, leucylleucine, danazol, and enalaprilat were significantly down-regulated (*p* < 0.05).

### 3.4 Molecular docking screening for metabolites that potentially possess anti-PCV2 effects

**Figure 3:**
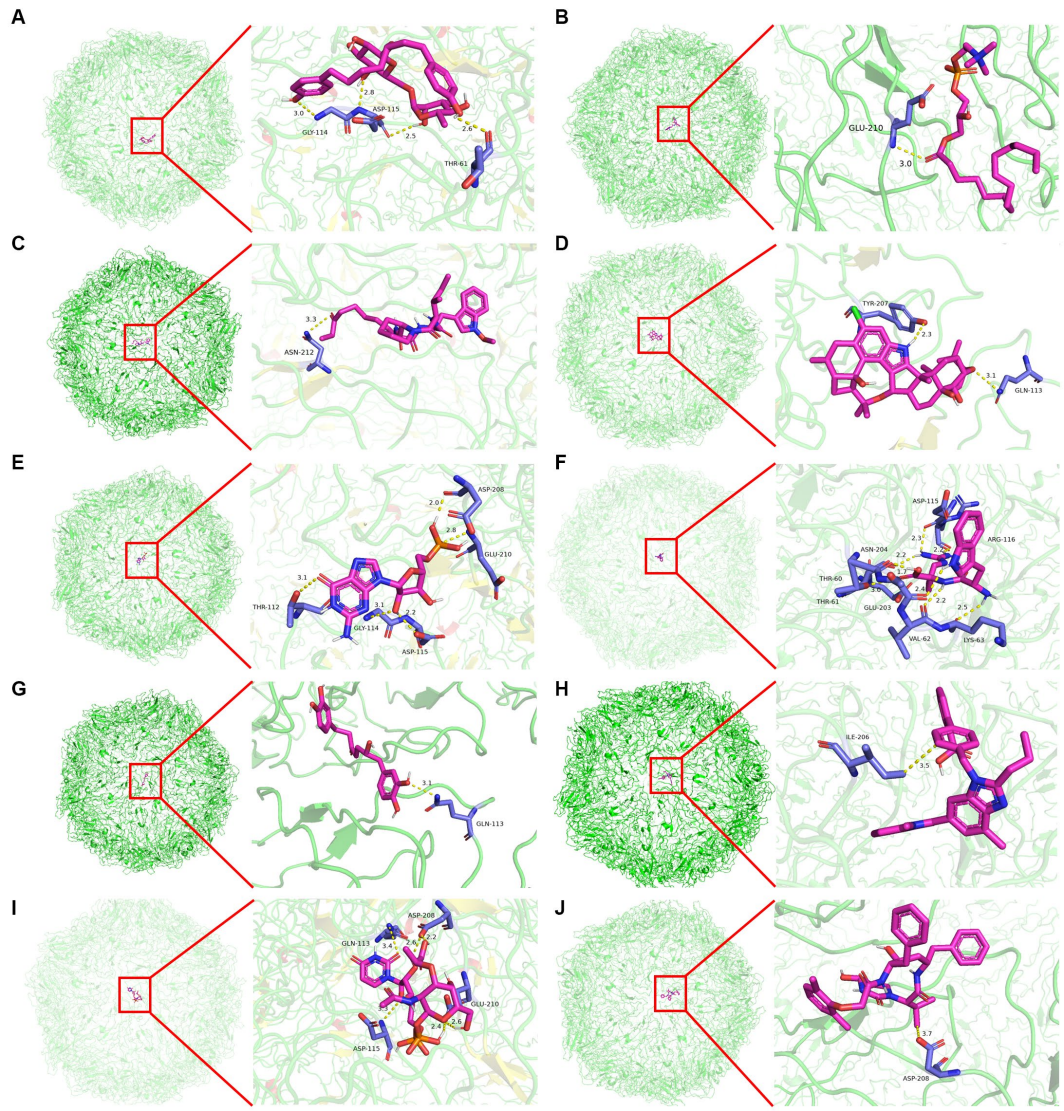
Binding pattern diagram of top 10 compounds in docking score ranking with Cap. **(A)** NCGC00381053-01; **(B)** Ergocristine; **(C)** Apicidin; **(D)** Penitrem a; **(E)** Guanosine 5′-monophosphate; **(F)** Trp-Arg; **(G)** 1,7-bis(3,4-dihydroxyphenyl)-6-hydroxyheptan-3-one; **(H)** Telmisartan; **(I)** UDP- N-acetylmuraminate; **(J)** Lopinavir Metabolite.

**Figure 4:**
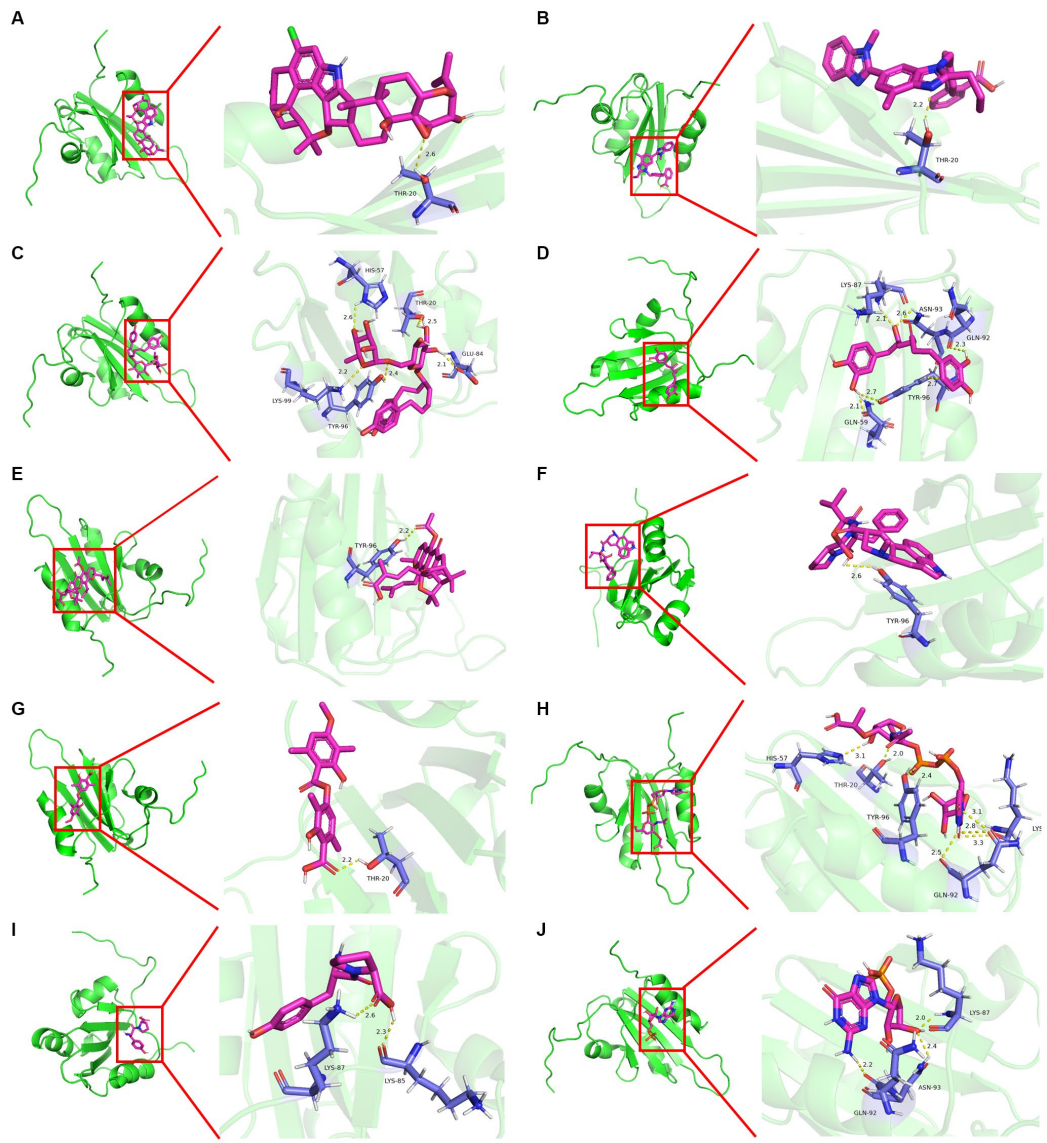
Binding pattern diagram of top 10 compounds in docking score ranking with Rep. **(A)** Penitrem a; **(B)** Telmisartan; **(C)** NCGC00381053-01; **(D)** 1,7-bis(3,4-dihydroxyphenyl)-6- hydroxyheptan-3-one; **(E)** Acetyl isogambogic acid; **(F)** Ergocristine; **(G)** Barbatic acid; **(H)** UDP-N-acetylmuraminate; **(I)** Tyr-Pro; **(J)** Guanosine 5′-monophosphate.

Molecular docking was conducted using UCSF DOCK6 software to elucidate the mode of action of Cap and Rep along with bacterial metabolites at the molecular level. The 128 compounds previously mentioned were docked into the binding pockets of Cap and Rep, and the top 10 compounds were selected based on their docking scores (Table 2). To create a binding pattern map of the compounds with Cap and Rep, the PyMOL Molecular Graphics System software was employed. The results indicated that NCGC00381053-01, ergocristine, penitrema, guanosine 5 'monophosphate, 1,7-bis (3,4-dihydroxyphenyl) -6-hydroxyheptan-3-one, UDP-N-acetylmuraminate, telmisartan, and other compounds could form potential complexes by establishing hydrogen bonds with Cap (Figure 3) and Rep (Figure 4).

**Table 2:** The scoring results for the molecular docking of Cap, Rep, and the compounds.

Number	Molecule name	Docking score (kcal/mol)	
		Cap	Rep
1	NCGC00381053-01	−8.63	−8.73
2	Ergocristine	−8.38	−7.99
3	Apicidin	−8.08	−6.58
4	Penitrem a	−7.99	−9.66
5	Guanosine 5′-monophosphate	−7.79	−7.63
6	Trp-Arg	−7.78	−7.08
7	1,7-bis(3,4-dihydroxyphenyl)-6-hydroxyheptan-3-one	−7.77	−8.37
8	Telmisartan	−7.69	−8.9
9	UDP-N-acetylmuraminate	−7.50	−7.79
10	Lopinavir Metabolite	−7.48	−7.17
11	Indinavir	−7.40	−6.95
12	Dorrigocin B	−7.37	−7.21
13	Deoxythymidine 5′-phosphate (dTMP)	−7.34	−6.45
14	Bilirubin	−7.32	−7.53
15	14-benzoylaconine	−7.30	−6.31
16	Resibufogenin	−7.27	−7.32
17	Succinate	−7.23	−6.09
18	Arg-his	−7.23	−7.24
19	Pro-Ala-Arg	−7.21	−6.6
20	Kanamycin a	−7.20	−6.54
21	Tyr-Gly	−7.20	−7.14
22	Microcystin lr	−7.09	−5.39
23	Asn-Ala-Arg	−7.07	−6.17
24	Arg-glu	−7.00	−5.92
25	Gly-Tyr	−6.98	−7.27
26	Cytidine 5′-monophosphate	−6.93	−6.66
27	Repaglinide acyl-.beta.-d-glucuronide	−6.92	−7.55
28	Barbatic acid	−6.88	−7.87
29	Manumycin a	−6.85	−6.27
30	Pro-Ala-Lys	−6.84	−5.88
31	Tyr-Pro	−6.82	−7.65
32	Ser-Glu	−6.78	−5.7
33	Gly-Pro-Arg	−6.75	−5.64
34	Gly-Phe	−6.73	−6.4
35	Benzoylnorecgonine	−6.72	−6.66
36	Mianserin n-oxide	−6.72	−7.52
37	(.+/−.)-cannabichromene	−6.71	−7.43
38	Gly-His	−6.67	−5.59
39	Phe-pro	−6.49	−6.98
40	Homoharringtonine	−6.48	−6.72
41	DL-tyrosine	−6.48	−5.92
42	Gly-Glu	−6.47	−5.45
43	Pro-Phe	−6.47	−6.74
44	Fructose	−6.45	−5.09
45	Vitamin c	−6.42	−6.41
46	Pseudouridine	−6.42	−5.26
47	Gln-gln	−6.40	−5.73
48	Aal toxin tb	−6.39	−5.26
49	Acetyl isogambogic acid	−6.39	−8.18
50	Hydroxyphenyllactic acid	−6.36	−6.83
51	D-allose	−6.35	−5.72
52	Met-Asp	−6.24	−5.24
53	Pro-Pro-Lys	−6.22	−5.31
54	Pro-Ala	−6.22	−5.78
55	2-hydroxy-6-methylquinoline-3-carbaldehyde	−6.21	−6.73
56	D-arabinose	−6.19	−4.77
57	Arginine	−6.13	−6.43
58	L-Arginine	−6.13	−5.26
59	Andrastin c	−6.13	−5.26
60	Esculetin	−6.11	−6.74
61	Xanthine	−6.11	−5.1
62	Arachidonoyl ethanolamide phosphate	−6.10	−5.79
63	Guanine	−6.06	−5.43
64	Posaconazole	−6.01	−6.11
65	D-glutamine	−6.00	−5.19
66	Phenyllactic acid	−6.00	−6.22
67	2’-O-Methyluridine	−5.97	−5.95
68	Enniatin b	−5.95	−4.48
69	Val-Pro	−5.94	−6.2
70	Porphobilinogen	−5.94	−5.47
71	Carbamic acid	−5.88	−7.54
72	(S)-2-Hydroxyglutarate	−5.87	−5.16
73	Ala-Pro-Lys	−5.85	−5.43
74	Glutamine	−5.83	−5.14
75	Glu-Val	−5.81	−5.49
76	His-Pro	−5.81	−6.06
77	D-Ribose	−5.79	−5.21
78	Hydroxyisocaproic acid	−5.76	−5.05
79	Baccatin iii	−5.75	−5.08
80	Hypoxanthine	−5.74	−6.52
81	2,8-quinolinediol	−5.74	−4.7
82	Etofenprox	−5.65	−7.37
83	Malate	−5.63	−4.78
84	Benazolin	−5.62	−6.07
85	N6-methyladenine	−5.62	−4.84
86	Glycerol 3-phosphate	−5.61	−4.62
87	1-stearoyl-2-hydroxy-sn-glycero-3-phosphate	−5.51	−5.22
88	L-Arabinono-1,4-lactone	−5.51	−6.61
89	5-nitroquinolin-8-ol	−5.51	−5.18
90	Carvedilol	−5.48	−7.12
91	Gly-Ala	−5.47	−5.08
92	Adenine	−5.33	−5.04
93	(r)-2-hydroxystearic acid	−5.30	−5.04
94	N-acetylserine	−5.29	−4.62
95	Geldanamycin	−5.28	−5.92
96	Dihydrothymine	−5.25	−4.98
97	Ile-Pro	−5.24	−5.48
98	Lys-Pro	−5.23	−5.42
99	Valine	−5.22	−5.76
100	1-palmitoyl-2-oleoyl-phosphatidylglycerol	−5.22	−4.96
101	1-pentadecanoyl-sn-glycero-3-phosphocholine	−5.21	−5
102	L-Norleucine	−5.15	−4.79
103	Thymine	−5.13	−4.41
104	Guanidoacetic acid	−5.13	−4.71
105	Methylmalonic acid	−5.13	−4.87
106	1,2-dipalmitoyl-sn-glycero-3-phospho-(1′-rac-glycerol)	−5.09	−5.09
107	Ricinoleic acid	−5.05	−5.63
108	Linolenic acid	−5.04	−4.92
109	Cytosine	−5.03	−4.46
110	Gly-Val	−5.02	−5.17
111	Proline	−5.00	−4.58
112	Methionine sulfoxide	−5.00	−4.59
113	Benzoic acid	−4.99	−5.74
114	2-phenylacetamide	−4.99	−5.66
115	Acetylglycine	−4.95	−4.37
116	KDdiA-PC	−4.89	−4.8
117	Schizandrin a	−4.87	−4.63
118	Glyceric acid	−4.85	−4
119	Indole	−4.63	−5.69
120	Taurine	−4.62	−3.91
121	Dl-lactate	−4.61	−4.02
122	Alanine	−4.60	−3.96
123	1,2-diamino-2-methylpropane	−4.14	−4.36
124	Methyl hexadecanoate	−4.11	−4.59
125	3-methyl-2-buten-1-ol	−4.09	−4.26
126	Propionic acid	−3.79	−3.87
127	Pyruvaldehyde	−3.44	−3.16
128	Valinomycin	2.98	−4.65

### 3.5 Joint analysis of Cap and Rep molecular docking results

**Figure 5:**
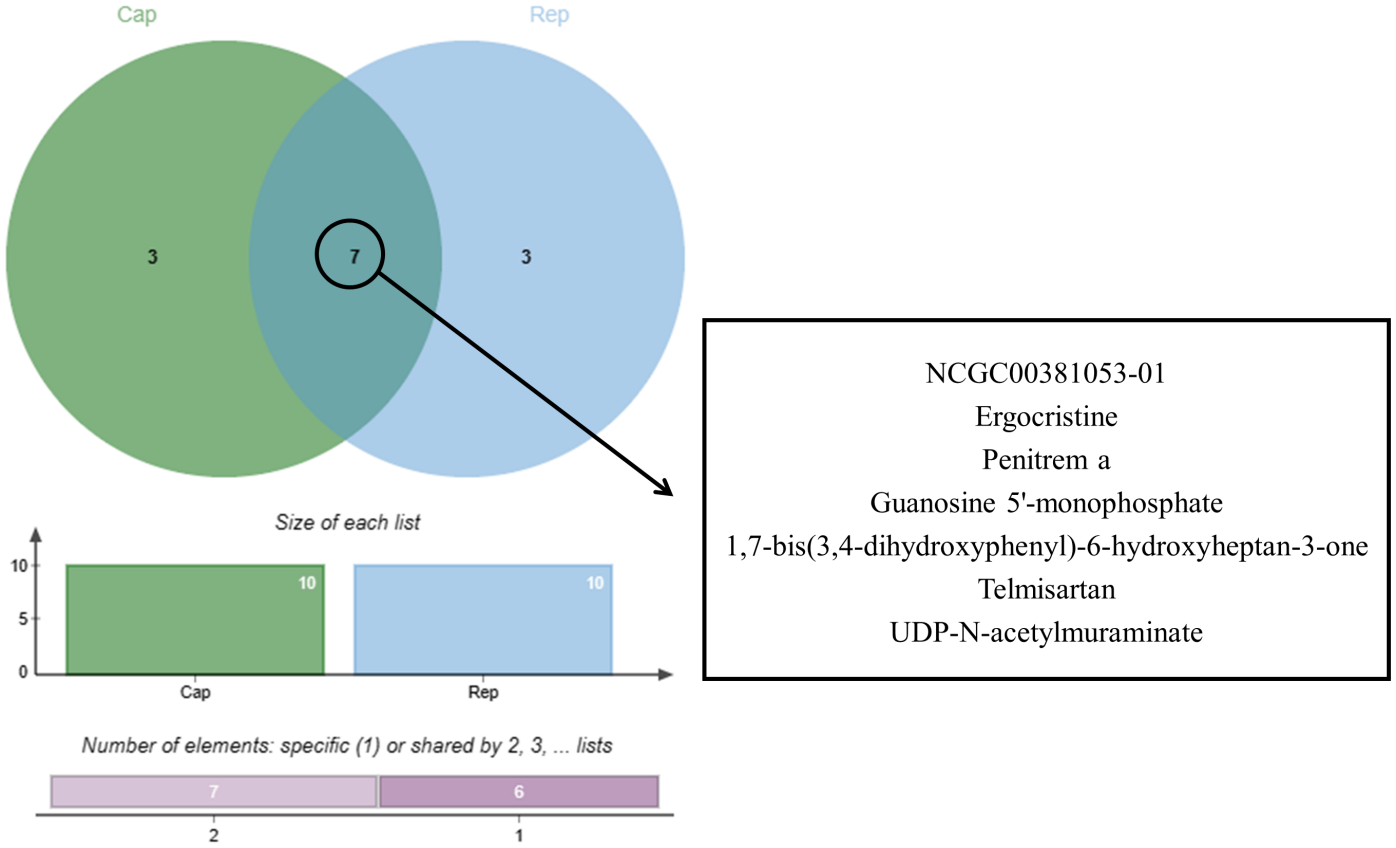
Joint analysis results of Cap and Rep molecular docking.

Bioinformatics analysis was conducted on the 10 compounds with the highest docking scores for Cap and Rep. The results suggested that *L. acidophilus* might have an anti-PCV2 role through seven of both top 10 compounds, including NCGC00381053-01, ergocristine, 1. 7-bis (3,4-dihydroxyphenyl) -6-hydroxyheptan-3-one, penitrema, guanosine 5 'monophosphate, telmisartan, UDP-N-acetylmuraminate (Figure 5).

## 4 Discussion

The pig industry in China and globally has suffered significant economic losses due to PCV2. our research group’s preliminary investigation revealed that matrine exhibited pharmacological effects against PCV2, both *in vitro* (Sun et al., 2015) and *in vivo* (Sun et al., 2020). This finding holds promise for developing a preventative and regulatory strategy for PCV2. Specifically, our study identified matrine’s anti-PCV2 function via modulating intestinal *L. acidophilus* (Cao et al., 2023). In addition, our previous study revealed that matrine promoted the proliferation of *L. acidophilus* in the intestinal tract of mice, leading to an increase in metabolites such as glutamine and Dl-lactate in feces. Although the precise anti-PCV2 mechanism of *L. acidophilus* remains elusive, numerous studies have demonstrated that probiotics mainly manifest antiviral and other pharmacological effects through their metabolites, such as butyrate (Lu et al., 2021), acetic acid (Antunes et al., 2019), and glucan (Nácher-Vázquez et al., 2015). This suggests that *L. acidophilus* may exert anti-PCV2 effects through its metabolites. Therefore, in this experiment, non-targeted metabolomics was implemented to screen for the changes in metabolites in the culture supernatant of *L. acidophilus* after 24 h of proliferation *in vitro*, and high-throughput molecular docking was then used to screen for the compounds with anti-PCV2 effect in the metabolites of *L. acidophilus*, to explore whether *L. acidophilus* exerts anti-PCV2 effect through its metabolites.

As a member of the Lactobacillus family of Gram-positive bacilli, *L. acidophilus* is widely acknowledged and recognized as a potent probiotic for the small intestine. Numerous investigations have demonstrated that *L. acidophilus* has anti-inflammatory (Li S. C. et al., 2019; Li W. Q. et al., 2019), anti-oxidation (Zhang et al., 2021), anti-tumor (Lau et al., 2024), improves hypertension (Romão da Silva et al., 2020), improves cardiovascular disease (Chen et al., 2013), protects intestinal mucosal barrier (Lightfoot et al., 2015), and a wide variety of other pharmacological effects. The results of non-target metabolomics showed that compared with the 0 h group, telmisartan, vitamin C, glutamine, carvedilol, Dl-lactate, and other substances in the culture supernatant of *L. acidophilus* increased significantly after 24 h of proliferation *in vitro*. Among these, the changes in metabolite levels of glutamine, Dl-lactate, and other compounds were consistent with the results of previous mouse experiments. Vitamin C, scientifically referred as ascorbic acid, is a potent antioxidant that exerts antitumor impacts and cellular shield against oxidative damage (Lykkesfeldt et al., 2014; Li S. C. et al., 2019; Li W. Q. et al., 2019); Telmisartan is a highly selective angiotensin II receptor (AT1) antagonist with a good antihypertensive effect, which is widely used in the treatment of essential hypertension in adults (Ontarget Investigators et al., 2008); Carvedilol has a good therapeutic effect on patients with chronic heart failure (Poole-Wilson et al., 2003); Glutamine is an amino acid drug that has protective and reparative outcomes on gastrointestinal mucosal injury (Decker-Baumann et al., 1999). The pharmacological effects of the compounds in the metabolites of *L. acidophilus* may correspond to the pharmacological effects of *L. acidophilus* such as anti-inflammatory, anti-oxidation, and anti-tumor, suggesting that *L. acidophilus* can exert the aforementioned pharmacological effects via its metabolites.

The results of molecular docking in this study showed that the docking scores of telmisartan, glutamine, and other compounds with PCV2 Cap and Rep were negative. A multitude of investigations have demonstrated that during ligand-receptor binding, conformations of the binding are more stable when docking scores are lower. This suggests that compounds such as telmisartan and glutamine, among others, might possess anti-PCV2 properties. Telmisartan is a cardiovascular-related disease treatment drug, but some recent studies have shown that it has antiviral effects. Tripathi et al. (2020) used surface plasmon resonance technology to prove that telmisartan can be used as a potential inhibitor of the replication of Chikungunya virus (CHIKV). De et al. (2022) confirmed that telmisartan limits the infection of CHIKV through the AT1/PPAR-γ/MAPKs pathway *in vivo* and *in vitro* models. Dhaka et al. (2023) found that telmisartan can inhibit the replication of the SARS-CoV-2 virus. Glutamine is the most prevalent free amino acid in serum. Ren et al. (2013a,b) found that dietary supplementation of L-glutamine can boost the immune response and eliminate PCV2 in mice. Liu et al. (2018) found that glutamine deficiency can promote PCV2 infection by activating the Ros-mediated JAK2/STAT3 signaling pathway, indicating that glutamine has a certain inhibitory effect on PCV2 proliferation. In addition to its anti-PCV2 effects *in vivo*, *L. acidophilus* has the ability to enhance glutamine and other metabolites both *in vivo* and *in vitro*. Furthermore, their potential anti-PCV2 function was validated by molecular docking data, implying that metabolites such as glutamine and telmisartan may be the key substances of *L. acidophilus* against PCV2. Additionally, a study by Misinzo et al. (2006) revealed that heparan sulfate and chondroitin sulfate B glycosaminoglycans on the cell surface may act as general receptors for PCV2, facilitating the virus’s attachment to host cells and subsequent entry. Moreover, our findings indicate that heparan sulfate could form complexes by establishing hydrogen bonds with Cap at residues Gly-144, Glu-210, Asp-208, Thr-122, and Gln-133. Chondroitin sulfate B could form potential complexes by establishing hydrogen bonds with Cap at residues Gly-114, Asp-115, Glu-210, and Gln-113(Data were not shown). The same docking sites were also found in the interactions between *L. acidophilus* metabolites and Cap. For example, glutamine could establish potential complexes with Cap at residues Asp-115, Glu-203, Tyr-207, Ser-205, and Gln-113. This overlap indicates that metabolites from *L. acidophilus* may exert anti-PCV2 effects by potentially interfering with the binding of PCV2 to cell surface receptors.

In conclusion, following a 24-h period of *in vitro* proliferation, a substantial alteration was observed in the metabolome of the culture supernatant containing *L. acidophilus*. This suggests that *L. acidophilus* may play an anti-PCV2 role through metabolites such as telmisartan. In addition, the pharmacological effects of *L. acidophilus*, such as anti-inflammatory, anti-oxidation, anti-tumor, blood pressure improvement, cardiovascular disease improvement, and protection of the intestinal mucosal barrier are likely associated to its metabolites. This experiment has provided insights into the changes in the metabolome caused by the proliferation of *L. acidophilus in vitro*, establishing a substantial study database for understanding the pharmacological mechanisms of *L. acidophilus* against PCV2, as well as its anti-inflammation and anti-oxidation properties.

## 5 Conclusion

After the proliferation of *L. acidophilus in vitro* for 24 h, the content of 128 compounds including Dl-lactate in the supernatant increased significantly. *L. acidophilus* may exert anti-PCV2, anti-inflammatory, anti-oxidation, anti-tumor, and improve hypertension and other pharmacological effects through its metabolites, such as telmisartan and Vitamin C.

## Data availability statement

The datasets presented in this study can be found in online repositories. The names of the repository/repositories and accession number(s) can be found in the article/Supplementary material.

## Author contributions

ZC: Conceptualization, Methodology, Writing – original draft, Writing – review & editing. XL: Data curation, Methodology, Software, Writing – review & editing. AH: Data curation, Validation, Writing – review & editing. PS: Formal analysis, Validation, Writing – review & editing. HZ: Supervision, Writing – review & editing. WY: Formal analysis, Writing – review & editing. KF: Visualization, Writing – review & editing. HY: Methodology, Writing – review & editing. ZZ: Software, Writing – review & editing. JZ: Formal analysis, Validation, Writing – review & editing. YS: Resources, Writing – review & editing. NS: Funding acquisition, Supervision, Writing – review & editing. HL: Funding acquisition, Project administration, Supervision, Writing – review & editing.
