# Supplementary material for: Analysis of the anti-PCV2 mechanism of Lactobacillus acidophilus based on non-target metabolomics and high-throughput molecular docking
Source: Front Microbiol. 2024 May 27;15:1416235. doi: 10.3389/fmicb.2024.1416235 (PMC11163031; doi:10.3389/fmicb.2024.1416235)
Supplement: Supplementary file 1 [file Table_1.pdf]

Supplementary Material

1 Supplementary Tables

Supplementary Table S1. Identification of differential metabolites in the supernatant of *L. acidophilus.*

Number	Name	VIP	FC	*P*	m/z	rt(s)
1	UDP-N-acetylmuraminate	1.04	145.25	0.000	678.09	451.70
2	1,2-dipalmitoyl-sn-glycero-3-phospho-(1'-rac-glycerol)	1.03	127.14	0.014	721.50	89.27
3	2-hydroxy-6-methylquinoline-3-carbaldehyde	8.14	47.27	0.000	186.05	238.97
4	1-palmitoyl-2-oleoyl-phosphatidylglycerol	1.21	34.45	0.018	747.52	39.90
5	L-Arabinono-1,4-lactone	17.58	22.02	0.001	147.05	116.44
6	D-allose	2.76	19.10	0.000	179.04	60.34
7	Phenyllactic acid	37.85	17.86	0.000	165.06	119.81
8	Acetylglycine	1.32	17.24	0.000	116.03	313.25
9	(r)-2-hydroxystearic acid	3.61	13.15	0.000	299.26	62.98
10	Mianserin n-oxide	2.80	9.81	0.006	561.30	401.04
11	Succinate	3.64	9.09	0.001	117.03	114.46
12	Enniatin b	1.30	8.99	0.000	640.41	319.12
13	Pro-Pro-Lys	3.34	8.94	0.000	341.22	538.96
14	Hydroxyphenyllactic acid	2.87	8.63	0.003	181.05	195.08
15	Carvedilol	1.09	8.04	0.000	407.19	163.32

16	5-nitroquinolin-8-ol	1.74	7.88	0.000	189.05	240.81
17	N-acetylserine	4.52	6.85	0.000	146.05	313.25
18	Microcystin lr	1.16	5.88	0.003	496.27	313.26
19	Hydroxyisocaproic acid	13.25	5.87	0.000	131.07	138.64
20	2'-o-methyluridine	4.10	5.76	0.000	257.08	91.29
21	Barbatic acid	1.17	5.38	0.003	359.10	195.16
22	Glycerol 3-phosphate	2.53	4.96	0.000	153.00	282.77
23	D-Ribose	1.62	4.36	0.000	209.07	160.32
24	Arg-his	1.42	4.17	0.021	312.19	291.19
25	Dl-lactate	10.37	4.12	0.013	89.03	239.49
26	SCHEMBL682260	4.31	4.06	0.034	530.29	243.42
27	D-glutamine	1.64	4.01	0.007	130.05	384.30
28	Dihydrothymine	1.24	3.62	0.000	127.05	381.62
29	Deoxythymidine 5'-phosphate (dTMP)	1.03	3.51	0.000	321.05	425.49
30	Adenine	13.78	3.35	0.000	136.06	169.81
31	Met-Asp	1.10	3.28	0.022	247.07	305.06
32	Pro-Ala-Lys	1.59	3.28	0.000	315.20	448.15
33	Glutamine	2.29	3.15	0.008	145.06	382.13
34	1,7-bis(3,4-dihydroxyphenyl)-6-hydroxyheptan-3-one	1.25	3.12	0.002	345.12	122.37

35	N6-methyladenine	1.77	3.07	0.000	148.06	133.72
36	Asn-Ala-Arg	1.33	2.98	0.000	360.19	302.26
37	His-Pro	2.11	2.92	0.000	253.13	352.68
38	Lopinavir M-3/M-4	3.05	2.88	0.010	627.37	306.88
39	1-stearoyl-2-hydroxy-sn-glycero-3-phosphate	2.58	2.86	0.000	437.27	240.48
40	Guanidoacetic acid	1.35	2.81	0.000	116.03	262.28
41	Guanosine 5'-monophosphate	1.44	2.58	0.009	364.06	474.54
42	Ala-Pro-Lys	1.81	2.57	0.027	315.20	468.64
43	Cytosine	5.17	2.57	0.009	112.05	204.98
44	Methionine sulfoxide	2.60	2.43	0.000	164.04	378.25
45	D-arabinose	4.56	2.40	0.000	149.05	159.68
46	Hypoxanthine	6.01	2.40	0.000	137.04	178.79
47	2,8-quinolinediol	1.60	2.37	0.012	202.07	70.17
48	Methyl hexadecanoate	2.81	2.34	0.007	315.25	102.75
49	Esculetin	6.01	2.25	0.003	177.04	216.81
50	Phe-pro	6.29	2.23	0.017	263.14	245.49
51	NCGC00381053-01	3.11	2.20	0.001	631.27	459.54
52	Val-Pro	5.48	2.16	0.021	213.12	270.56
53	Lys-Pro	1.46	2.07	0.010	244.16	432.92
54	Malate	2.86	2.06	0.000	133.01	414.48
55	Tyr-Pro	3.75	2.04	0.035	277.12	281.75

56	Cytidine 5'-monophosphate	1.40	2.04	0.003	322.04	468.76
57	Geldanamycin	1.02	2.01	0.006	583.28	400.95
58	Porphobilinogen	1.58	1.98	0.034	225.10	355.64
59	Fructose	1.12	1.97	0.028	179.06	240.12
60	Ile-Pro	6.88	1.90	0.025	229.15	253.21
61	Manumycin a	1.64	1.87	0.000	533.27	466.22
62	Xanthine	2.79	1.85	0.000	151.03	224.64
63	Gly-Phe	3.33	1.80	0.000	221.09	271.50
64	Bilirubin	1.26	1.72	0.001	583.27	409.38
65	Glyceric acid	1.60	1.71	0.009	105.02	319.00
66	MDL28170	1.10	1.69	0.004	765.39	440.44
67	L-Norleucine	1.04	1.67	0.001	263.19	271.89
68	Pseudouridine	1.17	1.64	0.000	243.06	251.32
69	Baccatin iii	1.21	1.62	0.001	587.26	437.23
70	Vitamin C	1.52	1.61	0.000	115.00	414.10
71	Schizandrin a	1.06	1.60	0.023	439.19	310.26
72	Apicidin	1.28	1.59	0.003	662.32	473.11
73	Guanine	2.31	1.57	0.005	152.06	238.05
74	Gly-Val	4.26	1.57	0.000	173.09	316.45
75	Gly-Tyr	1.51	1.53	0.001	237.09	313.23

76	Gly-Glu	2.26	1.53	0.000	203.07	432.32
77	(S)-2-Hydroxyglutarate	1.23	1.51	0.000	147.03	403.48
78	3-methyl-2-buten-1-ol	1.01	1.51	0.004	69.07	284.55
79	Gln-gln	1.48	1.49	0.000	273.12	375.67
80	Pro-Phe	1.09	1.48	0.001	227.11	431.54
81	Indinavir	3.02	1.43	0.000	612.33	315.18
82	(.+/-.)-cannabichromene	5.78	1.41	0.048	313.24	87.91
83	Andrastin c	1.04	1.41	0.000	457.24	263.73
84	Penitrem a	1.49	1.40	0.001	558.25	423.65
85	Fa 18:1+1o	3.01	1.40	0.034	297.24	55.43
86	Resibufogenin	1.35	1.39	0.004	429.21	366.00
87	1-pentadecanoyl-sn-glycero-3-phosphocholine	1.13	1.39	0.001	526.30	429.04
88	Thymine	3.76	1.38	0.001	125.04	77.86
89	Pro-Ala	1.46	1.37	0.004	185.09	347.53
90	Pro-Ala-Arg	1.94	1.37	0.001	343.20	310.88
91	Posaconazole	1.45	1.36	0.000	701.35	449.97
92	PKDdiA-PC	1.68	1.36	0.000	742.41	334.47
93	Gly-His	1.13	1.35	0.038	211.08	385.50
94	Linolenic acid	2.14	1.34	0.024	277.22	39.03
95	Repaglinide acyl-.beta.-d-glucuronide	1.24	1.32	0.000	627.27	424.65
96	Etofenprox	1.26	1.31	0.000	359.19	344.59

97	Kanamycin a	1.35	1.29	0.020	483.22	388.04
98	Tyr-Gly	1.09	1.29	0.001	237.09	289.79
99	Arachidonoyl ethanolamide phosphate	1.08	1.27	0.041	428.26	409.40
100	Gly-Ala	2.41	1.27	0.034	145.06	350.58
101	Ser-Glu	1.07	1.27	0.011	233.08	424.23
102	Gly-Pro-Arg	1.91	1.27	0.006	329.19	469.22
103	Telmisartan	1.93	1.27	0.021	469.24	341.46
104	Acetyl isogambogic acid	1.66	1.26	0.001	693.29	353.47
105	Benazolin	1.36	1.26	0.048	169.99	157.95
106	Arg-glu	1.45	1.24	0.000	304.16	461.56
107	Trp-Arg	1.10	1.23	0.000	361.18	467.85
108	Taurine	1.30	1.23	0.000	124.01	307.33
109	Glu-Val	1.89	1.22	0.008	245.11	386.69
110	14-benzoylaconine	1.33	1.22	0.013	604.30	371.63
111	Arginine	3.72	1.20	0.034	173.10	521.80
112	Aal toxin tb	1.32	1.20	0.000	528.31	429.29
113	L-Arginine	2.03	1.20	0.000	173.10	546.29
114	Valine	3.69	1.19	0.005	116.07	309.44
115	Homoharringtonine	1.60	1.18	0.000	546.29	455.28
116	Benzoylnorecgonine	1.11	1.18	0.013	549.21	450.95

117	Methylmalonic acid	2.59	1.17	0.015	117.02	398.71
118	Benzoic acid	1.98	1.17	0.007	123.04	310.62
119	1,2-diamino-2-methylpropane	2.62	1.17	0.003	72.08	311.26
120	Pyruvaldehyde	1.24	1.16	0.032	71.01	317.10
121	Propionic acid	1.03	1.14	0.003	73.03	398.82
122	2-phenylacetamide	2.98	1.14	0.018	136.07	310.62
123	Valinomycin	1.19	1.13	0.006	556.32	421.23
124	Ergocristine	1.45	1.11	0.012	610.28	447.86
125	Proline	2.34	1.11	0.048	116.07	322.01
126	Indole	1.02	1.11	0.037	116.07	330.62
127	DL-tyrosine	3.12	1.10	0.042	182.08	310.22
128	Alanine	2.00	1.09	0.045	88.04	356.70
129	ACon0_000485	1.04	0.86	0.018	486.20	264.86
130	Taurolithocholic acid sulfate	2.82	0.85	0.036	482.30	225.84
131	FC131	1.21	0.83	0.008	728.34	340.83
132	(z)-2-octylpent-2-enedioic acid	6.47	0.83	0.009	241.12	231.78
133	Arg-Val	1.33	0.82	0.011	274.19	388.83
134	Norethindrone 2,3,4-tri-o-acetyl-.beta.-d-glucuronide methyl ester	2.28	0.77	0.000	615.28	423.81
135	N-.alpha.-(tert-butoxycarbonyl)-l-lysine	1.18	0.77	0.004	247.14	216.78
136	N-acetyl-l-methionine	1.24	0.76	0.001	190.05	206.33

137	Phe-arg	2.60	0.76	0.000	322.17	216.69
138	Pantothenate	2.13	0.76	0.005	218.10	284.57
139	AZD4547	1.56	0.75	0.013	464.25	293.07
140	L-n5-(1-imino-3-pentenyl)ornithine	1.47	0.75	0.028	197.13	43.99
141	4',6'-dimethoxy-4-dimethylamino-2'-hydroxychalcone	3.22	0.75	0.002	328.14	312.84
142	Loggerpeptin a	1.19	0.74	0.000	1015.50	471.24
143	Isohernandezine	1.24	0.73	0.028	653.31	397.79
144	M-hydroxybenzoylecgonine	1.78	0.73	0.012	609.24	350.31
145	Rescinnamine	1.27	0.72	0.000	635.31	476.56
146	Desmethylcerivastatin	1.22	0.72	0.011	444.22	389.40
147	Leukotriene c4	1.69	0.71	0.000	626.30	443.99
148	Adenosine 3'-monophosphate	1.75	0.71	0.005	348.07	419.95
149	Curcumin	1.22	0.71	0.016	367.13	399.22
150	Beauvericin	1.42	0.71	0.016	806.41	319.82
151	N-Acetyl-D-lactosamine	1.71	0.70	0.047	383.14	269.83
152	Val-Cys-Arg	1.18	0.69	0.002	377.18	274.81
153	N-(2-phosphate-1r-methylethyl)-5z,8z,11z,14z-eicosatetraenamide	3.17	0.69	0.013	440.25	291.65
154	Adenosine 2'-monophosphate	2.13	0.69	0.010	346.05	418.89
155	1-oleoyl-sn-glycero-3-phosphocholine	2.05	0.69	0.016	556.31	215.47

156	1-(1,2-dioctanoylphosphatidyl)inositol	2.24	0.68	0.008	585.27	307.43
157	Cytidine	1.17	0.67	0.000	487.16	323.03
158	.alpha.-solanine	1.37	0.67	0.000	902.44	369.89
159	Arg-Gln	1.73	0.66	0.000	303.18	450.32
160	Palmitic acid	9.76	0.65	0.049	255.23	51.81
161	Etodolac	1.30	0.65	0.013	573.30	268.83
162	N-fructosyl isoleucine	3.70	0.64	0.002	292.14	324.17
163	Trp-Arg-Arg	1.92	0.62	0.000	517.28	402.36
164	Val-Leu	1.62	0.62	0.004	213.16	243.75
165	Terragine e	1.11	0.61	0.012	607.34	276.76
166	Tepraloxydim	1.33	0.61	0.003	364.12	431.52
167	Lys-Trp	1.40	0.60	0.001	333.21	414.82
168	alpha-N-Acetyl-L-glutamine	1.41	0.60	0.000	187.07	387.18
169	Reserpine	1.60	0.59	0.001	609.26	307.63
170	Ala-Tyr	1.06	0.59	0.022	251.10	291.03
171	YM 26567-1	1.05	0.59	0.002	547.28	378.36
172	Docetaxel	1.45	0.59	0.000	808.37	458.19
173	Arg-Ala	1.83	0.58	0.004	246.15	428.37
174	trans-2-Hydroxycinnamic acid	1.27	0.57	0.044	165.05	351.40
175	Pyruvate	4.77	0.57	0.005	87.01	141.52
176	Guanidinopropionic acid	1.05	0.57	0.000	130.05	264.21

177	Stevioside	2.30	0.56	0.003	827.37	298.11
178	Furostane base -2h + 1o, o-hex, o-pen-dhex	3.17	0.56	0.016	887.47	225.27
179	Val-leu-pro-val-pro	9.48	0.55	0.004	652.40	285.59
180	AKOS040738762	2.09	0.55	0.001	429.21	249.99
181	N-acetylmuramic acid	1.37	0.55	0.014	292.13	270.10
182	Fludrocortisone acetate	1.64	0.54	0.001	421.20	323.58
183	[5-[4-(3,4-dihydroxyphenyl)-2,3-dimethylbutyl]-2-hydroxyphenyl] 4-hydroxybenzoate	4.49	0.53	0.000	421.18	394.63
184	Citraconic acid	1.78	0.53	0.006	129.02	109.72
185	Leu-Lys	2.39	0.53	0.002	260.20	386.25
186	DL-proline	1.13	0.52	0.000	231.13	334.41
187	Val-Ser	1.92	0.52	0.000	205.12	306.03
188	Rebaudioside b	4.05	0.52	0.002	803.37	296.94
189	Val-Ala	3.87	0.52	0.005	189.12	272.44
190	Asn-Lys	1.06	0.51	0.003	261.15	450.47
191	Pro-leu	1.80	0.51	0.005	229.15	223.91
192	Val-Asn	3.36	0.50	0.002	230.11	320.94
193	Deoxyguanosine	2.70	0.49	0.000	152.06	271.77
194	Leu-Val	11.74	0.48	0.008	229.16	205.63
195	Dimerum acid [m-h2+fe]	2.35	0.46	0.000	509.24	353.41

196	Hoiamide a	1.39	0.46	0.000	926.44	279.98
197	Erucamide	3.02	0.46	0.040	338.34	36.50
198	Ile-Val	8.40	0.45	0.003	231.17	207.14
199	Tyr-Val	1.65	0.45	0.005	281.15	231.44
200	Phe-phe	1.53	0.44	0.021	313.15	163.87
201	L-gulono-1,4-lactone	6.17	0.44	0.021	177.04	119.08
202	Ltc4-[d5]	1.01	0.44	0.004	624.30	296.28
203	(19r)-9-acetyl-19-hydroxy-10,14-dimethyl-20-oxopentacyclo[11.8.0.0<2,10>.0<4,9 >.0<14,19 >]henicos-17-yl acetate	4.01	0.43	0.002	503.28	212.22
204	[d-asp3,e-dhb7]-microcystin-rr	2.44	0.42	0.001	1024.55	219.14
205	Lys-Leu	1.29	0.41	0.001	260.20	414.42
206	Val-Val	3.71	0.41	0.001	215.14	223.32
207	Tribenzylamine n-oxide	1.45	0.39	0.007	607.31	273.70
208	Polyphyllin b	1.85	0.38	0.003	1049.52	277.91
209	Heliotrine n-oxide	1.04	0.38	0.002	352.19	234.62
210	Enalaprilat	4.96	0.38	0.001	364.20	305.39
211	Leu-Gln	6.17	0.38	0.000	258.14	295.82
212	Phe-thr	1.16	0.37	0.001	267.13	221.99
213	Leu-Ser-Lys	1.52	0.37	0.001	347.23	399.10
214	Dibutyl phthalate	2.98	0.36	0.000	279.17	179.60

215	Ile-Asn	9.15	0.36	0.000	244.13	305.47
216	Leu-Arg	1.47	0.35	0.000	286.19	365.50
217	Inosine	2.14	0.35	0.001	267.07	225.27
218	alpha-Guanidinoglutaric Acid	1.09	0.35	0.000	231.11	243.63
219	Thymidine	6.35	0.31	0.000	241.08	104.47
220	Lys-Ile	1.70	0.30	0.003	260.20	363.62
221	2-keto-D-Gluconic acid	1.10	0.29	0.012	253.06	112.49
222	Leu-Trp	2.50	0.27	0.001	318.18	190.83
223	Tyr-His	1.27	0.27	0.000	319.14	316.22
224	7-hydroxy-4'-methoxyflavone	2.59	0.26	0.000	267.09	174.34
225	Alpha-ketoisovaleric acid	2.46	0.26	0.000	115.04	79.93
226	S4:18(p3:16/f1:2)	1.36	0.26	0.003	695.31	33.29
227	Ile-Thr	5.63	0.25	0.000	233.15	248.05
228	Met-His	1.11	0.25	0.000	287.11	313.48
229	Apigenin 7,4'-dimethyl ether	1.23	0.25	0.000	297.08	160.17
230	Ser-Phe	2.36	0.25	0.002	253.12	256.28
231	Leu-Gly-Leu	1.84	0.25	0.000	302.21	216.28
232	L-alanyl-l-norleucine	2.56	0.24	0.000	203.14	271.20
233	L-abrine	15.96	0.23	0.000	217.12	276.41
234	Leu-Ser	5.73	0.23	0.000	219.13	277.40

235	D-Arabinono-1,4-lactone	2.58	0.22	0.000	207.05	104.90
236	Cyclizine	3.33	0.22	0.000	265.15	192.84
237	Asn-Met	1.33	0.22	0.000	264.10	305.61
238	Val-Phe	4.17	0.21	0.000	263.14	190.56
239	Ile-Ala	4.92	0.21	0.000	203.14	245.13
240	Leu-Thr	8.47	0.20	0.000	231.13	246.71
241	Tyr-Ile	2.76	0.19	0.000	295.16	213.27
242	Tyr-Leu	3.25	0.17	0.000	293.15	211.57
243	His-Leu	6.20	0.16	0.000	269.16	308.37
244	Met-Ala	1.62	0.16	0.000	221.09	252.96
245	His-Lys	3.36	0.16	0.000	284.10	271.79
246	Camp	1.01	0.16	0.000	330.06	278.06
247	Leu-Phe	4.85	0.15	0.000	277.15	177.15
248	2-chloro-2'-hydroxy-4'-methylbenzophenone	2.33	0.15	0.006	245.03	89.33
249	Uracil	13.60	0.14	0.000	111.02	89.10
250	Glycyl-L-leucine	3.62	0.14	0.000	187.11	246.63
251	Ala-phe	1.88	0.14	0.000	237.12	217.38
252	Phe-Ala	2.26	0.14	0.000	235.11	215.50
253	Phe-met	1.55	0.13	0.000	297.13	180.45
254	HMB-Val-Ser-Leu-VE	5.03	0.13	0.002	506.30	238.70
255	Leucylleucine	9.69	0.13	0.000	245.18	193.17

256	Leu-Ala	6.66	0.12	0.000	201.12	243.77
257	Ile-Leu	13.23	0.12	0.000	243.17	191.06
258	Danazol	1.61	0.11	0.000	338.20	204.39
259	Tyr-Ser	10.92	0.10	0.000	268.10	200.57
260	Phenylpyruvate	2.26	0.08	0.000	163.04	58.08
261	Ile-Arg	4.86	0.08	0.000	288.19	243.57
262	Adenosine	21.91	0.08	0.000	268.10	178.23
263	Val-Met	3.18	0.07	0.000	249.13	212.34
264	Bursin	8.60	0.07	0.000	340.20	302.57
265	Leu-Met	2.92	0.06	0.000	263.14	196.25
266	Cyclic gmp	1.46	0.05	0.000	346.05	334.60
267	Guanosine 3',5'-cyclic monophosphate	2.10	0.05	0.000	344.04	333.34
268	.epsilon.-caprolactam	4.35	0.04	0.000	227.17	187.93
269	S-methyl-5'-thioadenosine	18.07	0.03	0.000	298.10	108.45
270	Niacinamide	3.84	0.01	0.000	123.05	67.62
